# Case Report: A case of heart failure recovery after non-ischemic cardiomyopathy following chimeric antigen receptor T-cell therapy

**DOI:** 10.3389/fcvm.2025.1608404

**Published:** 2026-01-08

**Authors:** G. Spears, J. C. Henson, S. Vellanki, A. Trikannad, H. Paydak

**Affiliations:** 1 College of Medicine, University of Arkansas for Medical Sciences, Little Rock, AR, United States; 2 Department of Internal Medicine, University of Arkansas for Medical Sciences, Little Rock, AR, United States; 3 Department of Internal Medicine, Willis Knighton Medical Center, Shreveport, LA, United States; 4 Myeloma Institute, University of Arkansas for Medical Sciences, Little Rock, AR, United States; 5 Division of Cardiology, University of Arkansas for Medical Sciences, Little Rock, AR, United States

**Keywords:** bundle branch block reentrant tachycardia, CAR-T therapy, cytokine release syndrome, heart failure, nonischemic cardiomyopathy, tachyarrhythmia, ventricular tachycardia

## Abstract

**Introduction:**

Chimeric Antigen Receptor T-cell (CAR-T) therapy has emerged as a highly promising immunotherapy for cancer treatment. Intensive research is being conducted focusing on adverse effects that CAR-T infusions may have due to the development of cytokine release syndrome (CRS). We aim to highlight a noteworthy case of ventricular tachycardia presented in the setting of acute non-ischemic cardiomyopathy following CRS after CAR-T therapy, along with providing a detailed discussion of subsequent inpatient and outpatient management.

**Case presentation:**

A 78-year-old man with a past medical history of prostate adenocarcinoma, hypertension, and type two diabetes mellitus, with no prior cardiac history or heart failure, underwent CAR-T therapy for diffuse large B-cell lymphoma (DLBCL). During the same hospitalization, he developed grade one CRS along with asymptomatic non-sustained ventricular tachycardia which was suspected to be bundle branch reentrant tachycardia (BBRT-VT). An echocardiogram performed after CAR-T infusion revealed a reduced left ventricular ejection fraction (LVEF) of 35%, a significant decline from his baseline LVEF of 51% two months prior. After the development of the ventricular tachyarrhythmia, amiodarone was initiated for rhythm control. However, over the subsequent five months, the patient had multiple hospital admissions for heart failure exacerbations. During this period, his EF declined to 15% when he was referred for implantable cardioverter defibrillator (ICD) implantation before gradually improving with continued amiodarone and guideline-directed medical therapy. Over the next 18 months, his LVEF recovered to 55%, highlighting the potential reversibility of immunotherapy-induced cardiomyopathy complicated by ventricular tachyarrhythmia.

**Conclusion:**

This case showcases a prolonged cardiac recovery following complications of CAR-T therapy. It also provides insight into the successful clinical management of a patient due to CAR-T-induced cardiomyopathy in the setting of potentially complex ventricular arrhythmias.

## Introduction

Chimeric antigen receptor T-cell (CAR-T) therapy is an important treatment of hematologic malignancies such as multiple myeloma, leukemias, and lymphomas resistant or refractory to initial chemotherapeutic regimens (1, 2). As CAR-T therapy approaches wider adoption in clinical practice, adequate identification of complications is vital to ensure patient safety (3). Adverse effects of treatment may potentiate complications resulting from an influx of pro-inflammatory cytokines such as interleukin-6 (IL-6), interferon-gamma (IFN-γ), and tumor necrosis factor-alpha (TNF-α) (4). These complications can manifest clinically through the development of cytokine release syndrome (CRS) and immune effector cell-associated neurotoxicity syndrome (ICANS).

Notably, CRS has been examined for its effects on cardiovascular function (5) with sinus tachycardia and hypotension being among the most common complications after CAR-T infusion (6). Development of arrhythmias has been implicated in up to 10% of CD-19 CAR-T infusions, some of which include ventricular arrhythmias (7–9). Severe cardiovascular events occur in up to 6.1% of patients after CAR-T treatment (8). Of patients that develop cardiomyopathies from CAR-T therapy, up to 50% have persistent systolic dysfunction past the conclusion of CAR-T treatment and resolution of CRS (9, 10). Ventricular arrhythmias can also develop in patients with preexisting conduction blocks secondary to the development of dilated cardiomyopathies (11, 12).

The following case details an instance of acute non-ischemic cardiomyopathy after CD-19 CAR-T therapy along with the codevelopment of a temporary ventricular tachyarrhythmia resulting in a protracted case of heart failure. We subsequently detail the patient's heart failure clinical course over the two years following CAR-T treatment and highlight the management strategies implemented to adequately address this complication and guide recovery from heart failure.

## Case presentation

We present the case of a 78-year-old man with a past medical history of hypertension, type two diabetes mellitus, gastroesophageal reflux disease (GERD), and previously diagnosed prostatic adenocarcinoma who was admitted for inpatient CD-19 CAR-T treatment in August 2022 for intra-abdominal diffuse large B-cell lymphoma (DLBCL) confirmed in 2021. At time of diagnosis, positron emission tomography-computed tomography (PET-CT) imaging of the chest, abdomen, and pelvis indicated metastatic involvement of the common iliac, external iliac, and retroperitoneal lymph nodes. Subsequent biopsy revealed CD-5/CD-10 negative DLBCL. Prior to CAR-T consideration, a treatment regimen of six cycles of chemotherapy consisting of rituximab, cyclophosphamide, doxorubicin, vincristine, and prednisone (R-CHOP) failed to achieve disease remission. Whole body PET-CT imaging approximately four months after R-CHOP therapy indicated an interval increase in size of the left common iliac, presacral, and external iliac lymph nodes. Similarly, whole body PET-CT seven months after R-CHOP therapy showed continued progression of disease by increased fluorodeoxyglucose (FDG) avid lymphadenopathy in the retroperitoneum, left pelvic sidewall, and left inguinal regions.

Before CAR-T therapy, multigated acquisition (MUGA) scanning was performed to establish baseline cardiovascular function (LVEF = 51%, Figure 2). Medications taken by the patient at the time of CAR-T initiation included leuprorelin and enzalutamide (concurrent management of prostate adenocarcinoma) and bridging chemotherapy regimen of rituximab, gemcitabine, and oxaliplatin (R-GemOX).

Early on day one of hospitalization, the patient developed a fever of 103.0 °F (39.4 °C), which persisted until hospital day three. He was diagnosed with CRS grade one and treated with tocilizumab, an interleukin-6 (IL-6) monoclonal antibody, to dampen the inflammatory response. Empiric antibiotic therapy was administered but discontinued after negative blood cultures. Due to an immune effector cell encephalopathy (ICE) score of 6/10 (component measure used to monitor for ICANS development, normal = 10/10) on hospital day two (deficits in knowing the year, month, standard sentence creation, and counting backward), workup for concern of ICANS grade two was performed. Magnetic Resonance Imaging (MRI) of the brain showed no evidence of any acute intracranial process and there were no clinical concerns for seizures or epileptiform discharges on electroencephalogram (EEG). After receiving one dose of tocilizumab, his fever resolved, and he remained afebrile for several days. On hospital days five and six, he briefly developed a secondary episode of CRS grade one with a fever of 101.3 °F (38.5 °C). Acetaminophen was administered and the patient became afebrile within twenty-four hours. Blood cultures collected again at fever recurrence were negative and the patient remained afebrile for the remainder of the hospitalization.

**Figure 1:**
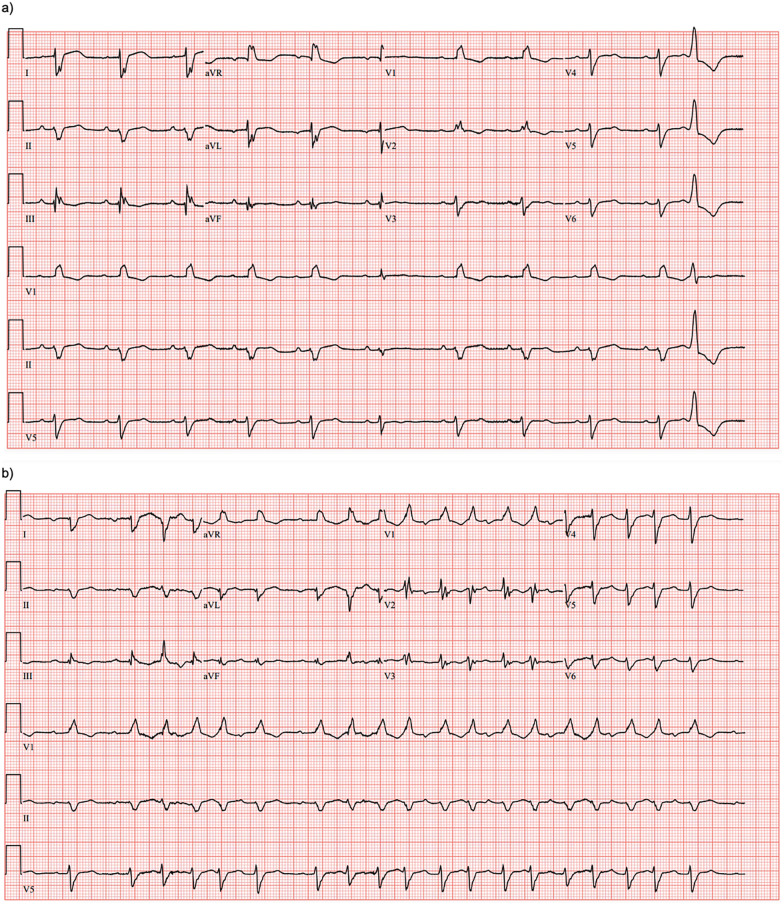
EKG collected one day before CAR-T infusion **(a)** showing sinus rhythm with PVCs and fusion complexes and RBBB. EKG following infusion **(b)** showing emergence of suspected BBRT-VT with AV dissociation and RBBB.

**Figure 2:**
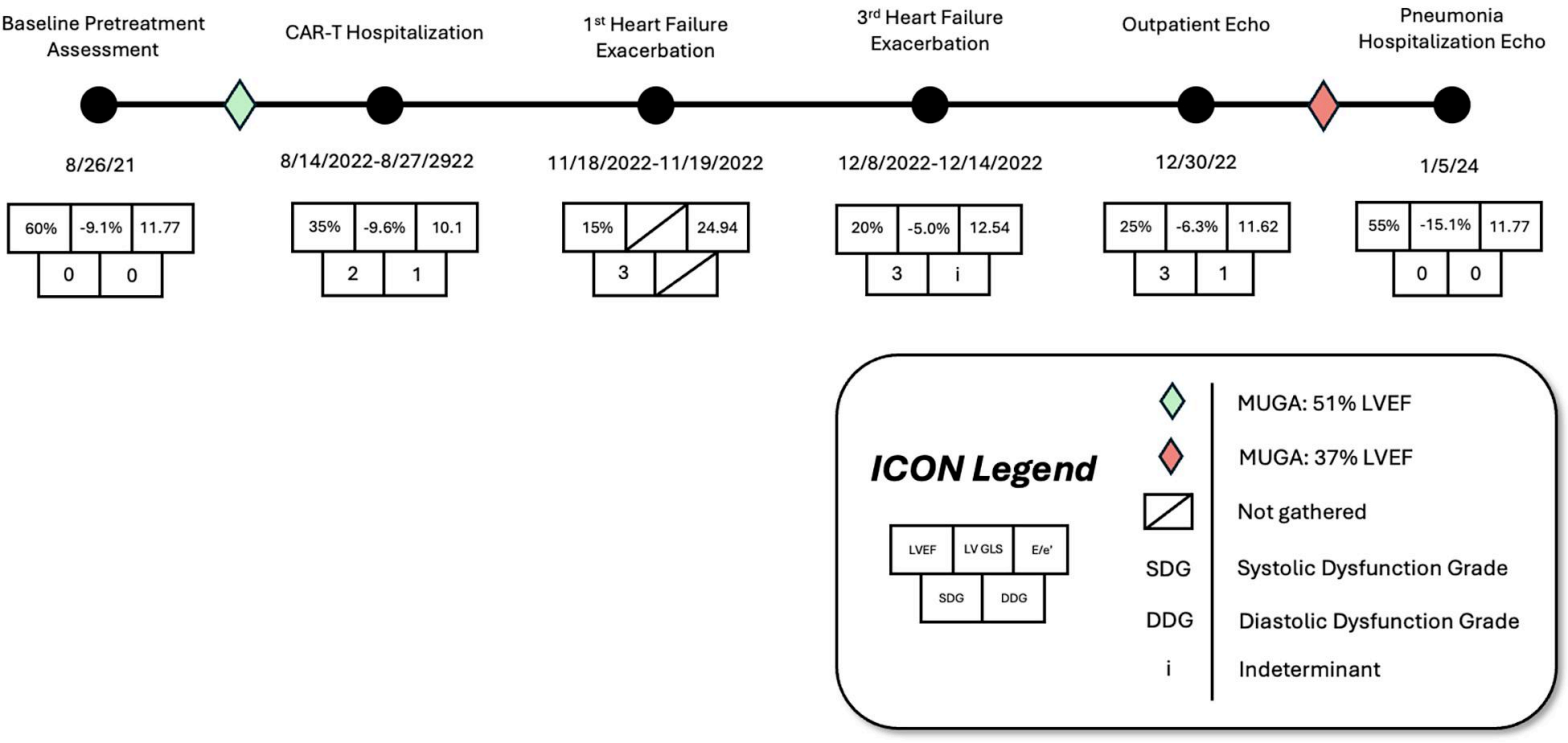
Timeline with associated cardiovascular imaging indices showing left ventricular ejection fraction (LVEF), global longitudinal strain (GLS), over the patient's treatment course prior to and following CAR-T infusion.

On hospital day two, the patient had an episode of non-sustained ventricular tachycardia (NSVT). Electrocardiogram (EKG) at that time showed sinus rhythm with 1st-degree AV block with fusion complexes. Transthoracic echocardiography (TTE) was performed, which showed a marked reduction in LVEF to 35% with mid-segment and basilar wall motion abnormalities; left ventricular wall thickness remained normal (Figure 2, Table 1). Serum troponins were not elevated during workup following infusion. Cardiology was consulted at this time due to concern of cardiomyopathy secondary to CAR-T treatment. On the fifth day of hospitalization, the patient began experiencing increased premature ventricular complexes (PVCs) on telemetry with further runs of NSVT on hospital days five through nine. On day eight, EKG recorded a period of ventricular tachycardia suspected to be right bundle branch reentrant tachycardia (BBRT-VT) with atrioventricular nodal (AV node) dissociation and concomitant right bundle branch and left posterior fascicular block (Figure 1b). We ultimately suspected this to be BBRT-VT with AV dissociation as evidenced by wide QRS morphology, presence of right bundle branch block (V2–V3), and regular interval spaced *p*-waves not associated with these subsequent QRS complexes. The presence of the right bundle branch block, indicating the direction of the reentrant tachycardia and AV disassociation are known hallmark feature of BBRT-VT (13–16) and indicates underlying His-Purkinje system disease precipitating this reentrant circuit. This EKG also indicates further evidence of underlying conduction system disease with concomitant left posterior fascicular block as demonstrated by rightward axis deviation, and an rS pattern in leads I and aVL and a qR pattern in leads III and aVF (Figure 1b) (17).

**Table 1:** Echocardiographic imaging showing GLS and apical four chamber view progression throughout clinical course.

Event	Global longitudinal strain analysis	Apical four chamber view
CAR-T hospitalization	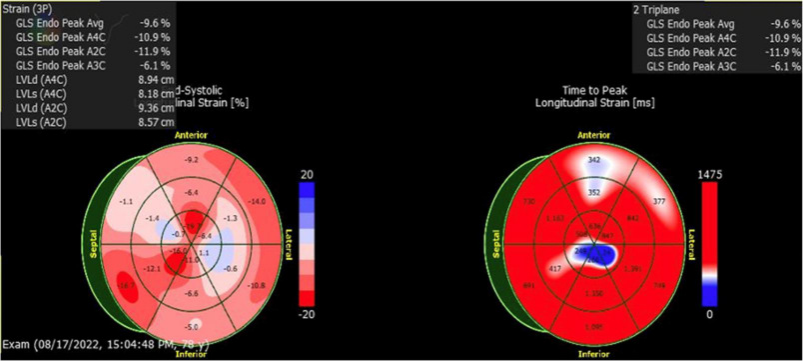	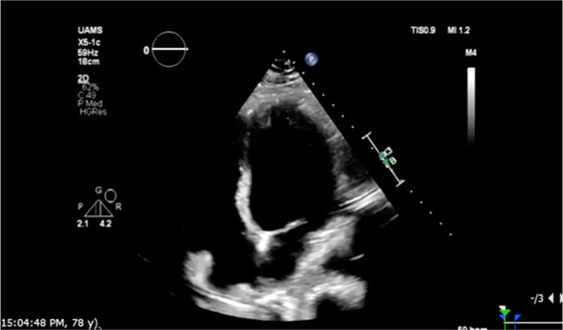
3rd heart failure exacerbation	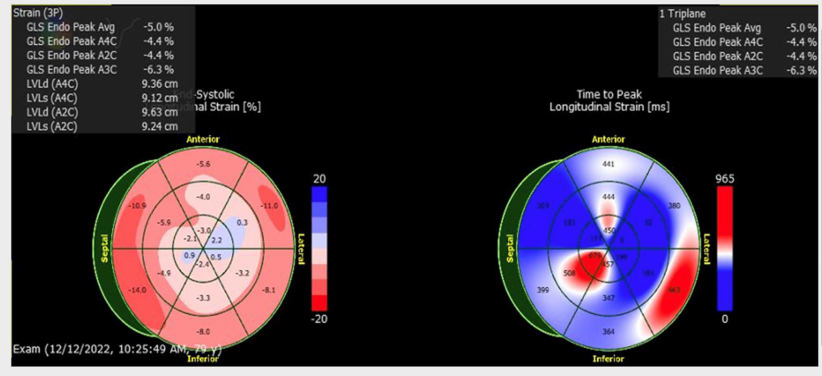	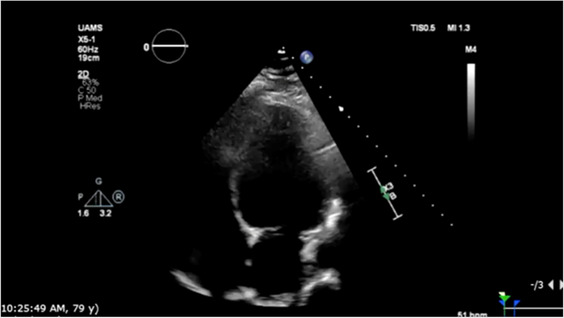
Pneumonia hospitalization	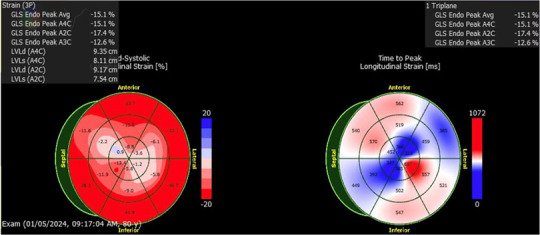	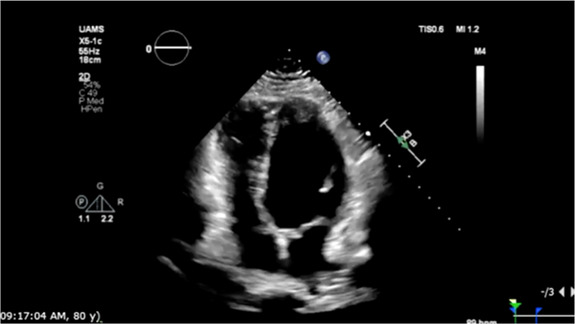

Repeat EKG later on hospital day eight showed a return to sinus rhythm with 1st degree AV block, right bundle branch block, and left posterior fascicular block. Amiodarone 400 mg was initiated on hospital day ten which prevented further episodes of ventricular tachyarrhythmias. Other differentials postulated by inpatient cardiology consultants due to initial inpatient echo findings included Tokutsubo cardiomyopathy given prior reports of development in patients following CAR-T cell infusions for DLBCL within five days after infusion (18). To further evaluate for potential ischemic etiology of new-onset cardiomyopathy, single positron emission computerized tomography (SPECT) imaging was obtained on hospital day eleven which showed heterogeneous uptake of radiotracer secondary to cardiomegaly without evidence of ischemia. LVEF calculated through SPECT imaging was 37% (Figure 2). Stress EKG also displayed no evidence of ischemia, with findings consistent with earlier baseline EKGs during admission. Because of the negative ischemia workup initially, combined with his deteriorating functional status, coronary angiography was considered too high-risk to rule out coronary ischemia. On hospital day thirteen, the patient was discharged with a new diagnosis of heart failure with reduced ejection fraction (HFrEF). EKG performed on the date of discharge showed no evidence of recurrence of ventricular tachyarrhythmias. Lisinopril 2.5 mg and Metoprolol Succinate 50 mg daily were started at that time for HFrEF guideline-directed medical therapy (GDMT), and Amiodarone 400 mg daily for rhythm control with cardiology outpatient follow-up and plans for GDMT up-titration as tolerated.

The following three-month clinical course was uneventful until late November 2022 when the patient was found to have acutely worsened lower extremity edema, shortness of breath, and orthopnea. The patient was briefly admitted for observation and a trial of IV diuretics overnight for a suspected heart failure exacerbation. Vitals on admission were HR: 91 bpm, BP: 155/113 mmHg, RR: 21/min, and SpO2 95%. BNP was 1,955.9 pg/mL. Initial EKG showed normal sinus rhythm with a 1st-degree AV block, persistent right bundle branch block and posterior left fascicular block as evidenced from prior. TTE showed a further reduction in LVEF estimated at 15%. Echocardiography also revealed concentric left ventricular hypertrophy with anteroapical akinesis, mild mitral regurgitation, and normal left ventricular size (Figure 2). Chest radiographs indicated fluid overload with bilateral increased lower lung thickening suggestive of acute pulmonary edema. The patient responded well to diuresis and the remainder of the hospitalization was uneventful. He was discharged 12 h after admission. The patient was initiated on oral Furosemide 40 mg daily at the time of discharge and further referred for continuing outpatient cardiology follow-up. Three days later, the patient was admitted again for a heart failure exacerbation. Vitals on admission were HR: 88 bpm, BP: 130/100 mmHg, RR: 18/min, and SpO2 99%. BNP was measured at 627 pg/mL. Chest radiographs again suggested fluid overload and EKG had similar findings as prior. No TTE was performed during this 3-day admission. At discharge, Furosemide 40 mg was switched to Bumetanide 1 mg daily.

Eleven days later the patient experienced another episode of decompensation requiring a third admission for heart failure exacerbation. Vitals on admission were HR: 85 bpm, BP: 171/119, RR: 30/min, and SpO2 92% with BNP measured at 3,291 pg/mL. Chest radiographs at this time showed pulmonary vascular congestion without pulmonary edema. EKG on admission showed sinus tachycardia with no interval change in 1st degree AV block, premature ventricular complexes, and a nonspecific intraventricular conduction delay. The patient was given IV Furosemide 40 mg BID for four days with subsequent uneventful recovery. TTE showed a marginal increase in LVEF to 20% with severe left ventricular systolic dysfunction and grade II diastolic dysfunction with severe diffuse left ventricular hypokinesis (Figure 2, Table 1). At discharge, Lisinopril 2.5 mg was discontinued and Sacubitril-Valsartan 24–26 mg was initiated while continuing home Bumetanide 1 mg daily and Metoprolol 50 mg daily. SGLT-2 inhibitor therapy (Empagliflozin 10 mg daily) was also added to the patient's GDMT. The patient's Amiodarone dosage was reduced to 200 mg daily from 400 mg daily for optimized rhythm control.

Following the third discharge for heart failure exacerbation, no further admissions for heart failure occurred. The patient attended outpatient cardiology follow-up via telemedicine in January of 2023 with in-person follow-up in April of 2023. Discussions with both the patient and family members involved with his healthcare in clinic also allowed for the development of optimal GDMT adherence and pharmacological rhythm control during this time. Given the significant frequency of recent admissions, the patient was considered as a candidate for an implantable CardioMEMS device, however, the patient declined and medical management alone was continued. Repeated measurements of liver function tests, thyroid function tests, as well as pulmonary function tests showed no evidence of amiodarone toxicity. MUGA scanning obtained five months later later in the spring of 2023 showed an increase in LVEF to 37% (Figure 2). Of note, around this time, the patient developed a chronic CAR-T infusion-related B-cell aplasia, resulting in two hospitalizations for neutropenia/hypogammaglobulinemia and sepsis requiring vasopressor support, respectively. BNP obtained during the first of these hospitalizations was 134 pg/mL. Serial TTEs obtained during both admissions revealed improvement of LVEF to 55% with normalization of diastolic function (Table 1). EKGs revealed similar findings as described above. The patient has continued on a regimen of Metoprolol 50 mg, Sacubitril-Valsartan 24–26 mg, Empagliflozin 10 mg, Bumetanide 1 mg, and Amiodarone 200 mg daily with no further events or admissions recorded in the medical record.

## Discussion

Chimeric Antigen Receptor (CAR) T-cell therapy has revolutionized the treatment of relapsed or treatment-refractory hematologic malignancies like that of diffuse large B-cell lymphoma (DLBCL). Despite its known success, CAR-T therapy is associated with significant cardiovascular risks, particularly cardiomyopathies complicated by ventricular arrhythmias (7, 9, 19). This is problematic for patients with pre-existing cardiovascular comorbidities, with up to one-third of cardiomyopathies noted to have developed following CAR-T in patients with previously established heart failure (20).

The mechanism of such acute cardiomyopathies is thought to relate to the surge in levels of cytokines such as IL-6, interleukin-8 (IL-8), tumor necrosis factor alpha (TNF-α), and interferon gamma (IFN-γ) that occur during CAR-T therapy, and act to both directly and indirectly impair cardiac function. IL-6, in particular, likely contributed to this acute presentation through a complex dynamic involving immune dysregulation, direct myocardial injury, and microvascular dysfunction. IL-6, in particular, is known to promote endothelial dysregulation resulting in capillary leakage (7, 20), propagate a sustained pro-inflammatory gene expression through STAT-3/NF-kB hyperactivation (21, 22), enhance maladaptive cardiac remodeling through ERK/JNK activation (23), and activate the complement system membrane attack complex (MAC) capable of lysing myocardial cells (24, 25), all of which may have contributed to the local effects of this acute cardiomyopathy. Furthermore, this cytokine storm may have contributed to systemic metabolic derangements producing a metabolic insufficiency causing reduced cardiac contractility. This in turn likely led to low cardiac output thereby exacerbating tissue hypoxia. Furthermore, this tissue hypoxia likely led to additional cytokine release creating a self-propagating cycle that further worsened cardiac function. Advanced studies such as cardiac MRI have proven to be useful in further assessing cardiac function under T2-mapping techniques in detecting myocardial edema associated with myocarditis; however, based on the initial cardiac workup, we believe that this would not have likely changed overarching management throughout the treatment course following infusion (26).

We also believe that this acute-onset cardiomyopathy could have precipitated the development of the ventricular tachyarrhythmia, as the patient had been monitored on continuous telemetry before the EKG-captured event without prior occurrence. This case is important in that the level of CRS required to elicit this level of cardiac dysfunction was lower than what is typically seen with most cardiomyopathies occurring with CRS grade ≥2 (20, 27). Mechanisms for the development of ventricular arrhythmias post CAR-T are often multifaceted. When combining pre-existing His-Purkinje conduction abnormalities with acute structural changes in the ventricular myocardium, alternative conduction circuits become more likely to form (28). These acute myocardial changes may induce scar formation secondary to inflammation, altering baseline conductance patterns and facilitating the development of macroreentrant ventricular arrhythmias with preexisting conduction blocks (6, 12, 29). Electrophysiological study during intracardiac electrogram (iEGM), can help distinguish rare arrhythmias like BBRT-VT from reentrant circuits secondary to myocardial scar formation (30). BBRT-VT is commonly seen in patients with significant structural heart disease and conduction system disease such as those with dilated cardiomyopathy (31) but can also be seen in patients with nonischemic cardiomyopathy and reduced left ventricular ejection fraction (13). Our interpretation, however, is limited given the absence of an electrophysiologic study confirming the reentrant circuit due to the patient's critically low LVEF and depressed functional status in the months following infusion. Due to the clinical deterioration of the patient and decline in functional status, invasive electrophysiologic study for confirmation of the arrhythmia was also considered to be too high risk compared to the apparent improvement with pharmacological rhythm control and GDMT alone.

Treatments for these ventricular tachyarrhythmias often include radiofrequency catheter ablation (RFCA) once the reentrant circuit is identified (32). Even patients with severe functional impairment (New York Heart Association class III and IV) may still be candidates for ablation to deal with such arrhythmias (33). Due to the acuity of the patient, a precipitous drop in LVEF and functional HFrEF status, and the resolution of the ventricular arrhythmia on subsequent EKG on hospital day eight, it was proposed that our patient benefited most from the rapid implementation of amiodarone to prevent the recurrence of the suspected macroreentrant circuit. While rhythm control through amiodarone aided in the control of the patient's arrhythmia, rapid GDMT administration remained critical to preventing lasting cardiac dysfunction as a result of the precipitating cardiomyopathy (34). The use of diuretics, beta-adrenergic antagonists, and SGLT-2 inhibitor medications likely aided in preventing further cardiac remodeling (35, 36), allowing the significant yet prolonged full recovery of both systolic and diastolic cardiac function. Additionally, the gradual recovery of this patient's LVEF and symptomatic relief is also suspected to be due, in part, to better compliance with GDMT achieved through shared decision-making in the clinic setting.

Proper monitoring during CAR-T therapy is critical to detect and manage potential cardiovascular complications. If complications develop, post-treatment follow-up is equally important, with recommended assessments on day seven, including EKG, echocardiography, and biomarker testing, to monitor cardiac function (8). High-risk patients should undergo follow-up at three months to assess for delayed cardiotoxicity, ensuring that any late onset issues are detected early (6). Echocardiograms performed before and after CAR-T initiation are crucial to establish baseline cardiovascular function and to assess for cardiotoxicity after CAR-T treatment respectively (37). Early indicators like lower baseline left ventricular global longitudinal strain (LV GLS) and higher baseline ratio of early mitral inflow velocity to early diastolic mitral annular velocity (*E*/*e*′) are associated with higher risks of cardiac events after CAR-T (38). Interval reductions in LVEF 10% or reduced left ventricular shortening fraction (LVSF) 5% may also be reliable markers for cardiac dysfunction in CAR-T patients (16). Our patient's baseline echo findings before CAR-T therapy did not appear to indicate definitive pre-existing cardiac dysfunction.

Even though no overt cardiovascular dysfunction was present before infusion, prior anthracycline exposure as a result of R-CHOP chemotherapy may have contributed to subclinical cardiovascular risk that helped manifest the post-infusion cardiac sequelae observed in this patient (10). The dramatic improvement in the patient's LVEF (15% at the lowest measurement weeks after infusion to 55% nearly 18 months later) additionally serves as an example to the benefits awarded to prompt GDMT administration and pharmacologic rhythm control when more invasive techniques carry excessive risk. Lastly, the case serves as a reminder to the clinical impact that shared decision-making can have when GDMT and rhythm control regimens are optimized and better adherence is obtained.

Although heart failure and the development of tachyarrhythmias following CAR-T therapy are commonly reported complications, the co-development of both chronic cardiomyopathy and ventricular tachycardia coupled with a substantial recovery in LVEF with GDMT and initial pharmacological rhythm control is notable. Further studies examining ventricular tachyarrhythmias that develop following CAR-T therapy would allow for the development of better treatment guidelines and benefit further clinical decision-making through the establishment of proper treatment paradigms. As the field of Cardio-Oncology is rapidly expanding with the implementation of many novel immunotherapies, the elucidation and association of specific cardiotoxicities resulting from immunotherapy is paramount to better guide the clinical management of these patients.

## Conclusion

While CAR-T therapy remains a groundbreaking treatment for many hematologic malignancies, it introduces unique challenges regarding cardiovascular health. This case represents an acute ventricular arrhythmia presenting with the onset of a chronic cardiomyopathy secondary to CRS following CAR-T therapy. The successful recovery of LVEF with early GDMT initiation and pharmacological rhythm control underscores the importance of early recognition and aggressive management of both HFrEF and ventricular arrhythmias in CAR-T patients. Further investigation is needed to further elucidate the specifics of CAR-T-related acute cardiomyopathies, to develop cardiac monitoring protocols for these patients, and to establish standardized treatment paradigms when complications such as the one described in this case occur.

## Data Availability

The original contributions presented in the study are included in the article/Supplementary Material, further inquiries can be directed to the corresponding authors.
